# Hooping Cough

**Published:** 1846-10

**Authors:** 


*Hooping Cough.*—At a meeting of the Medical Society of London, held Jauuary, 1845, Dr. Waller stated that he had recently employed the extract of belladonna, in two cases of hooping cough with the best results. He gave it, in the dose of one-twelfth of a grain, three times a day to a child four years of age. In these cases there was simply the spasmodic cough, without any indication whatever of the existence of inflammation. Mr. Crisp remarked, that in the majority of cases, he had found the antiphlogistic plan of treatment to be the one best adapted to the early stages of hooping cough. He viewed the disease generally as inflammatory, or at all events congestive. Contrasting the antiphlogistic treatment (leeches followed by blister and the use of tartar emetic) with that which he had formerly pursued, he had every reason to be satisfied with the change he had made. The prussic acid he had usually found to be of service only for a day or two.

Dr. Wiltshire had treated, at the Infirmary for Children, simple uncomplicated hooping cough with two or three emetics of ipecacuanha—one every morning or second morning—followed, for two or three days, with nauseating doses of antimony, and had found this plan of great service in the early stages of the disease. Hemlock and ipecacuanha were of benefit afterwards in relieving the cough. With respect to belladonna, he was fearful of its employment, for notwithstanding the evidence in its favor, adduced by some of the German physicians, others had found that this medicine had a tendency to increase vascular action in the brain, and to produce hydrocephalus.

Dr. Chowne laid particular stress, in the treatment of hooping cough, on the necessity of keeping the patient in a warm temperature, and using every means to prevent his taking cold—nauseating doses of ipecacuanha, he considered to be frequently of benefit. With reference to the production of emphysema by hooping cough, he did not believe it possible.

Dr. Clutterbuck viewed the disease as one of a specific character, resulting from a specific cause—it was a specific inflammation of the bronchial membrane, but apt to induce inflammation of other organs, as the lungs or head, and it was these complications that constituted the dangerous character of hooping cough. He had little confidence in any remedy for this affection. The disease should be narrowly watched, with the view of prevention, rather than of active treatment—if mild, nothing was necessary to be done, it would terminate spontaneously—if inflammation of the brain, lungs, or other organs was threatened, antiphlogistic measures were demanded.

Dr. Golding Bird regarded hooping cough, in its first stage, as invariably an inflammation of the bronchial tubes, larynx and trachea, of a specific character, and implicating, in some peculiar manner, the par vagum. This inflammation lasted for a definite period, which is influenced by constitution and other causes. In its second stage, the disease is *nervous*—the specific irritation of the *par vagum* being kept up, altogether independent of inflammation; or when this is present it is merely accidental: the disease is afterwards kept up by the influence of habit. In the first stage the remedies for bronchitis (emetics, diaphoretics, the warm bath, and a warm temperature) are advisable. When the inflammatory stage is passed, attention should be directed to subdue irritation in the par vagum by narcotics—such as conium, in conjunction with carbonate of potash, hemlock, and hydrocyanic acid. Embrocations to the spine and chest are also useful. When bronchorrhœa becomes troublesome, small doses of alum, with sedatives, are of advantage. When the bronchorrhœa has ceased, tonics are indicated—the kind of tonic to be determined by the constitution of the patient. Emphysema does not occur as the consequence of hooping cough.

The president of the society had found in some cases where belladonna was given, that the poisonous, rather than the curative effects of that remedy developed themselves, even though the doses administered were remarkably small. With reference to the irritation of the par vagum in hooping cough, he related a case in which this nerve had become exposed, from the formation of an abscess, or other cause; and it was remarkable, that when the nerve was in contact with the air, a spasmodic cough resembling that of pertussis was produced: when the nerve was covered over by a cicatrix, the spasmodic congh ceased.

Dr. Golding Bird remarks (Guy's Hospital Reports, April, 1845,) that, in the second or nervous state of pertussis—after all inflammatory symptoms have subsided; and when, with a tolerably cool skin and clean tongue, the patient is still distressed by the more or less copious secretion of viscid mucus from the bronchi; each attempt to get rid of which, produces the exhausting and characteristic cough; no remedy will be found to act so satisfactorily, or to give such marked and often rapid relief to the child as alum. He has not yet met with any other remedy which is equally efficacious. Dr. Bird generally gives the alum in doses of from two to six grains in children of from one to ten years of age, repeated every four or six hours. For a child of two or three hours, he employs generally the following formula:

℞ Aluminis gr. xxv; extr. conii gr. xij; syrup. rhædos. ʒij; aq. anethi ℥iij. m. Dose a medium sized spoonful, every sixth hour. Dr. B. has never met with any inconvenient stringent effects on the bowels during its exhibition; on the contrary, in more than one instance it produced, he says, diarrhœa. The only obvious effects resulting from its use were, diminished secretion, and of a less viscid mucus, with a marked diminution in the frequency and severity of the spasmodic paroxysms.—*Trans. Phila. Col. Phys.*

